# Correction: Rapid room temperature synthesis of red iridium(iii) complexes containing a four-membered Ir–S–C–S chelating ring for highly efficient OLEDs with EQE over 30%

**DOI:** 10.1039/c9sc90213a

**Published:** 2019-10-01

**Authors:** Guang-Zhao Lu, Ning Su, Hui-Qing Yang, Qi Zhu, Wen-Wei Zhang, You-Xuan Zheng, Liang Zhou, Jing-Lin Zuo, Zhao-Xu Chen, Hong-Jie Zhang

**Affiliations:** State Key Laboratory of Coordination Chemistry, Jiangsu Key Laboratory of Advanced Organic Materials, Collaborative Innovation Center of Advanced Microstructures, School of Chemistry and Chemical Engineering, Nanjing University Nanjing 210093 P. R. China yxzheng@nju.edu.cn zuojl@nju.edu.cn zxchen@nju.edu.cn; State Key Laboratory of Rare Earth Resource Utilization, Changchun Institute of Applied Chemistry, Chinese Academy of Sciences Changchun 130022 P. R. China zhoul@ciac.ac.cn

## Abstract

Correction for ‘Rapid room temperature synthesis of red iridium(iii) complexes containing a four-membered Ir–S–C–S chelating ring for highly efficient OLEDs with EQE over 30%’ by Guang-Zhao Lu *et al.*, *Chem. Sci.*, 2019, **10**, 3535–3542.

This correction is being published to draw the readers’ attention to the authors’ closely related papers, published at a similar time in *Journal of Materials Chemistry C*^1^ and *Materials Chemistry Frontiers*,^2^ which should have been cited in this *Chemical Science* paper. The authors understand that they should have notified the journal’s editors about the related manuscripts when this *Chemical Science* paper was under review.

All three papers report cyclometalated iridium(iii) complexes that contain a four membered ring based on the same Ir–S–C–S backbone. However, the complexes reported in each paper have different cyclometalated moieties and/or different dithiocarbamate derivatives as the main ligands and ancillary ligands, respectively.

In this *Chemical Science* paper, three complexes were reported with 4-(4-(trifluoromethyl)phenyl)quinazoline as the main ligand and three dithiocarbamate derivatives as ancillary ligands. In ref. 1, two complexes were reported with the same main ligand but with two different ancillary ligands. In ref. 2, the authors reported five complexes using 1-(4-(trifluoromethyl)phenyl)isoquinoline as the main ligand and five dithiocarbamate derivatives as ancillary ligands.

Therefore, although all the papers reported iridium(iii) complexes with similar structures, these materials show different photophysical properties and device performances. However, ref. 1 and ref. 2 should have been cited in this *Chemical Science* paper.

The Royal Society of Chemistry apologises for these errors and any consequent inconvenience to authors and readers.
